# The Toxic Effects of Veronal

**Published:** 1907-07-13

**Authors:** 


### Points in Therapeutics.

### THE TOXIC EFFECTS OF VERONAL.

Veronal has proved itself to be an excellent hypnotic for ordinary cases of insomnia, also for febrile conditions, and even under certain circumstances for sleeplessness due to pain. As a rule the sleep comes on in about a couple of hours, it is deep and refreshing, but it is perhaps of rather shorter duration than that which follows some other hypnotics. There are seldom any feelings of heavy drowsiness the next day, and upon the whole the drug is one of the most satisfactory of the milder hypnotics. It acts more readily in women than in men.

There are, however, certain untoward effects which have occasionally followed the use of veronal. The commonest of these is delirium; in a certain small proportion of patients veronal acts as a deliriant rather than a hypnotic. It would seem, therefore, that veronal should be avoided when, from the nature of the illness itself, there is any tendency towards delirium; and it is decidedly unwise either to give it in large doses at one time, or to repeat an average dose at a comparatively short interval, in any case in which the patient's idiosyncrasy as regards veronal is not already known from previous experience. It sometimes happens that the desired sleep does not follow the first dose, and one is tempted to give a second a few hours after; it is better that the second dose should be something else than veronal unless one is quite sure that the particular patient takes veronal well. The usual dose is 7 grains, and it is inadvisable to exceed this. In the great majority of suitable cases where a simple hypnotic is required, refreshing sleep will follow, but it is well to know that there are a few cases—apparently suitable so far as one can tell beforehand—in whom the effect may be delirium instead of sleep.

There are already a few cases of suicidal poisoning by veronal on record, the doses which have proved fatal being a little over 1 drachm. The main symptom which results is coma, deepening to death. A few persons who have swallowed a lethal dose have been fortunate enough to vomit soon afterwards, in consequence of which they have evacuated a sufficient quantity of the poison to recover; the majority do not vomit, and recovery is impossible unless their stomachs are washed out quickly. The coma may persist for three days before death ensues, and meanwhile the face becomes very cyanosed, the respiration rate is slightly increased, and the pulse, well sustained for a time, becomes gradually more feeble and ultimately intermittent. The reflexes are not lost in the earlier stages of the coma, the pupils being of moderate size and reacting sluggishly to light, but later the reflexes get less and less, and are finally abolished altogether. One patient, a man aged 54, whose case is recorded by Zörnlaub, lived for twenty-four hours after taking 2½ drachms of veronal; another, a woman aged 26, died on the third day after taking 1½ drachms.

We have laid considerable stress upon the dangers of veronal; but we would at the same time repeat that in suitable cases and in suitable doses it is a pleasant and fairly certain hypnotic. It is on this very account that it is liable to become misused; it should never be taken except when specially advised, and patients should not be allowed to get into the way of taking a dose of it whenever they feel inclined. It is at present too easy to purchase successive doses of these hypnotics, until a fatal dose has been accumulated without any suspicions being aroused.

